# The Suinfort^®^ Semen Supplement Counters Seasonal Infertility in Iberian Sows

**DOI:** 10.3390/ani11113176

**Published:** 2021-11-06

**Authors:** Javier Piñán, Felipe Martinez-Pastor, Beatriz Alegre, Magdalena Maj, Roy N. Kirkwood, Juan Carlos Domínguez, Rodrigo Manjarín

**Affiliations:** 1 Institute of Animal Health and Cattle Development (INDEGSAL), Universidad de León, 24071 León, Spain; javierpinan@gmail.com (J.P.); ctibag@unileon.es (B.A.); jcdomt@unileon.es (J.C.D.); 2 Department of Molecular Biology (Cell Biology), Universidad de León, 24071 León, Spain; 3 Department of Animal Medicine, Surgery and Anatomy (Animal Medicine and Surgery), Universidad de León, 24071 León, Spain; 4 Department of Biological Sciences, California Polytechnic State University, San Luis Obispo, CA 93407-0255, USA; mmaj@calpoly.edu; 5 School of Animal and Veterinary Sciences, University of Adelaide, Roseworthy, SA 5371, Australia; roy.kirkwood@adelaide.edu.au; 6 Animal Science Department, California Polytechnic State University, San Luis Obispo, CA 93407-0255, USA; rmanjari@calpoly.edu

**Keywords:** Iberian pig, sow, artificial insemination, Suinfort^®^, seasonal infertility

## Abstract

**Simple Summary:**

Efficient pork production relies on a predictable supply of market pigs. Seasonal infertility caused by heat stress decreases fertility in sows during the summer months, impacting breeding targets and decreasing the efficiency of pork production. The present study examined the effect of a seminal additive containing caffeine, oxytocin, and lecirelin on the fertility and prolificacy of Iberian sows during two consecutive years. The results confirmed that inclusion of the additive in semen prior to AI decreased the seasonality effect, increasing the percentage of pregnant Iberian sows throughout the year.

**Abstract:**

Suinfort^®^, a commercial semen supplement demonstrated to increase fertility and litter size in commercial sows, was tested to improve reproductive performance in Iberian sows. A total of 1430 Iberian sows were artificially inseminated (AI) with semen from Duroc boars and assigned by parity to receive the seminal additive Suinfort^®^ containing 2 IU oxytocin, 5 µg lecirelin, and 2 mM caffeine (SF; *n* = 1713 AI), or to serve as non-supplemented controls (CON; *n* = 2625 AI). CON showed a lower fertility comparing to winter for spring (*p* = 0.001) and summer (*p* < 0.001); summer was lower than autumn (*p* = 0.012). SF removed this seasonal effect (*p* > 0.05). Fertility was significantly higher for SF sows during summer (*p* = 0.025) and autumn (*p* = 0.004). Total born, live-born, stillborn, and mummified piglets did not differ between CON and SF but were impacted by the season, with total and live-born decreasing in summer compared with autumn (*p* < 0.001) and winter (*p* = 0.005). In conclusion, seminal supplementation with Suinfort^®^ improved the fertility of Iberian sows during periods of seasonal infertility.

## 1. Introduction

The pig industry depends on artificial insemination (AI) with liquid semen doses from selected boars. This technique has increased efficiency and genetic improvement [1], yet it can reduce sows’ reproductive performance [2]. A significant factor contributing to suboptimal results from AI in sows is the lack of boar stimulation during semen deposition, which decreases myometrial contractions and uterine sperm transport [3]. As a result, fewer sperm reach the oviduct, decreasing the oviductal sperm reservoir, potentially resulting in lower fertility [4,5].

An additional challenge is the seasonal infertility of the sows. This seasonal effect is expressed in temperate climates, as a decrease in farrowing rate and prolificacy during the summer and early autumn [6,7,8]. The high temperatures affecting the reproductive function of the sows [9] are the leading cause of this seasonal effect.

We have shown that semen doses supplemented with oxytocin, cloprostenol, and buserelin increased litter size and fertility and helped to ameliorate seasonal infertility [8,10]. This work led us to develop Suinfort^®^, a commercial semen supplement containing oxytocin, lecilerin, and caffeine (Pat. no. WO2018/1002/14), which increases fertility and litter size following AI in sows [11]. Nonetheless, our previous trials only included typical commercial sows (Landrace × Large White) and, therefore, Suinfort^®^ remains to be tested in other breeds, particularly Iberian sows. Iberian pigs are reared in Spain for their cultural and gastronomic value and ability to adapt to harsh environments [12,13]. However, due to relatively poorer genetic selection, the reproductive performance of Iberian pigs is lower compared with other commercial breeds, and the sows might be more susceptible to seasonal infertility [13]. The average prolificacy for this breed is 7.5 piglets per farrowing (6.0 to 8.3, depending on the study, see [12] for a collection of studies on this topic).

Therefore, the objective of this study was to investigate whether Suinfort^®^ could be used to improve fertility in Iberian sows, particularly when bred in the hotter months typically associated with reduced reproductive performance.

## 2. Materials and Methods

### 2.1. Animals

The University of León Animal Care Committee reviewed and approved the protocol and procedures. The study was performed with Iberian sows located in a farrow-to-finish farm near Valverde de Mérida (38°54′ N, 6°13′ W, 267 m MSL, Badajoz, Spain) for two consecutive years (June 2016 to August 2018). Sows between 8 and 98 months old were housed in individual gestation stalls and had boar contact for 5–10 min/d for up to 15 d to facilitate detection of the first post-weaning estrus. They remained in the stalls after AI and until confirmed pregnant 35 d after mating. Pregnant sows were housed in pens with 38 sows/pen until they were moved to individual farrowing crates from 1 week before farrowing until weaning at 28 d. Coolers and artificial lighting kept farrowing room temperatures between 18 and 34 °C throughout the year with a minimum daily photoperiod of 12 h.

Semen was collected weekly from 68 Duroc boars (12–30 months of age) using routine protocols. The sperm quality was checked at the stud farm following standard protocols, with all having acceptable levels of motility (≥80%) and morphology (≥75% morphologically normal sperm and ≥95% normal acrosomes). Sows were artificially inseminated (caudal cervix) at estrus detection and 24 h intervals while exhibiting estrus with 3 × 10^9^ sperm in 80 mL extender. All semen doses were used within 48 h of collection. At estrus detection, sows were assigned by parity to receive the seminal additive Suinfort containing 2 IU oxytocin, 5 µg GnRH analog (lecirelin), and 2 mM caffeine (SF; *n* = 1713) or serve as non-supplemented controls (CON; *n* = 2625). In total, 4338 AI were carried out in 1430 sows (interquartile range: 2–5 AI per sow), with an average parity of 4.9 ± 2.8 (mean ± SD). The SF was included in the seminal dose 15 min before the first insemination only, as we have previously shown no additional effect of seminal supplement inclusion in the second insemination [14]. Sows went to term, and farrowing rates and subsequent litter sizes were recorded.

### 2.2. Data Analysis

Data analysis was performed in the R statistical environment v.4.0.4 [15]. Fertility (AI resulting in farrowing) and piglet counts were analyzed by generalized linear mixed-effects models (GLME; logistic regression for fertility and Poisson for counts) [16]. The fixed effects of the models included season, treatment, parity (as gilts, 1, 2–4, 5–10 and >10 farrowing groups, following [17]), and their interaction, and the random effects included sow and boar. Data are presented as means ± SD. Multiple comparisons were corrected with the Tukey post hoc test, and significant effects were considered at *p ≤* 0.05.

## 3. Results

**Figure 1:**
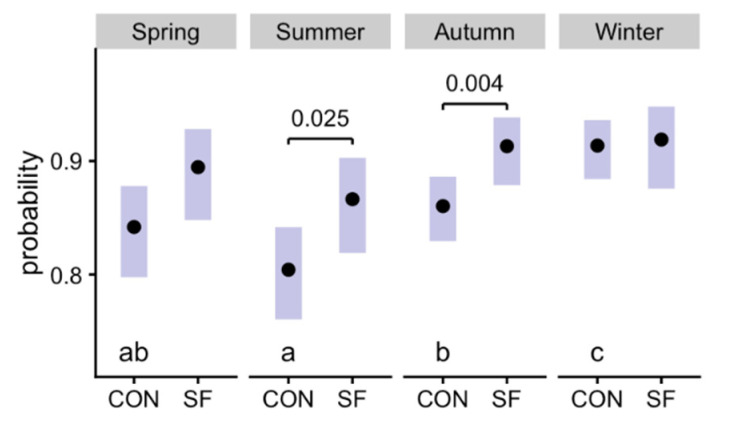
Effects of season and Suinfort^®^ on sow fertility (CON: Control; SF: Suinfort^®^). Summer and autumn negatively affected fertility (probability for a given sow to get pregnant), but the use of Suinfort^®^ reduced that effect. The plot shows estimated means (points) and their 95% confidence intervals (bars) for each treatment × season combination. Table 1 shows the number of observations in each group (inseminated sows). The effect of season was significant for the Control treatment (different letters, a, b, c, indicate *p* ≤ 0.05 among seasons for CON; season effect was not significant in the SF group). The effect of Suinfort^®^ was significant (*p* values shown) in summer and autumn, countering the seasonal effect.

Farrowing rates following AI (Figure 1; sample sizes in Table 1) were affected by the interaction between season and treatment (*p* = 0.05). Parity had a significant effect (*p* < 0.001; higher results in the 1, 2–4 and 5–10 groups compared with gilts, and 2–4 compared with 5–10 and >10 groups), with no interaction with the treatment. We observed a decrease in fertility for CON during spring and summer compared with winter (*p* = 0.001 and *p* < 0.001, respectively); there was some recovery by autumn (*p* = 0.012 with summer), although fertility remained significantly lower than in winter (*p* = 0.022). SF reduced this trend and was only the summer that was significantly lower than winter (*p* = 0.005). Comparing the groups within each season, fertility was significantly higher in the SF group during both summer (*p* = 0.025) and autumn (*p* = 0.004).

**Figure 2:**
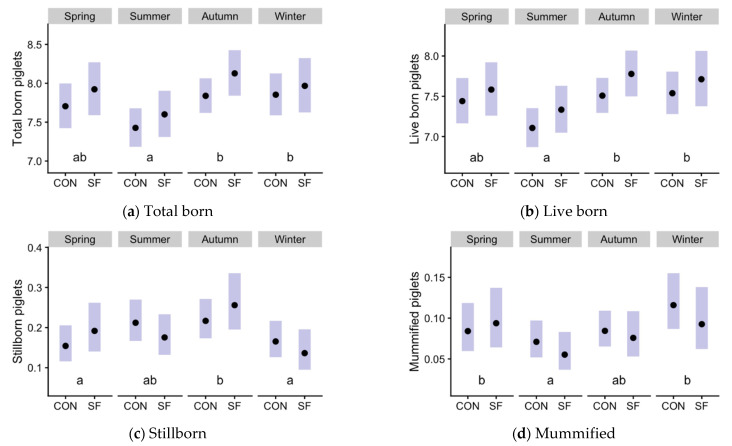
Effects of season and Suinfort^®^ in pig prolificacy (CON: Control; SF: Suinfort^®^). Estimated means (points) and their 95% confidence intervals (bars) for each treatment × season combination, for the numbers of total born (**a**), live-born (**b**), stillborn (**c**) and mummified (**d**) piglets in each farrowing. Table 2 shows the number of observations in each group (farrowed sows). The interactions between factors were not significant, and the effect of season was significant for all the variables (different letters a, b, indicate *p ≤* 0.05 among seasons). There were no significant differences between CON and SF groups.

For sows that farrowed, total born, live-born, stillborn, and mummified piglets (Figure 2; sample sizes in Table 2) did not differ between CON and SF, but these variables were impacted by season and parity. Total and live-born piglets (Figure 2a–d) decreased in summer compared with autumn (*p* < 0.001) and winter (*p* = 0.005 for total, *p* = 0.003 for live born). Stillborn piglets (Figure 2c) decreased in spring and winter compared with autumn (*p* = 0.011 and *p* < 0.001, respectively). Mummified piglets (Figure 2d) decreased in summer compared with spring (*p* = 0.036) and winter (*p* ≤ 0.001). Parity significantly affected total born and live-born with *p* < 0.001 (2–4 and 5–10 groups yielding more total and live piglets than gilts); stillborn and mummified with *p* = 0.010 (1-farrowing group with lower numbers). The Supplementary Materials show the distributions of the counts for each category by season and treatment (Figures S1 and S2).

## 4. Discussion

The present data demonstrate the effect of season on Iberian sow fertility, with both farrowing rates and litter sizes being lower in summer and autumn. The effects of season on the fertility of typical commercial sows have been reviewed, and it was suggested that both temperature and photoperiod are involved in the seasonal effects on sow fertility [9]. One link between the lower farrowing rates and smaller litters could be the quality of the ovulated oocytes and the subsequent corpora lutea. Interestingly, ovarian follicular progesterone concentrations are lower in the summer than in the winter [18]. Moreover, an attenuated LH surge could adversely affect follicular luteinization with resultant impaired corpora luteal function in the summer [9], and the lower follicular progesterone would support this suggestion. Although the endocrinology of estrus and early pregnancy in Iberian sows remains to be determined, it is reasonable to assume that they would be physiologically similar to commercial genotypes. If this is accepted, then the methods to counter seasonal effects on the hypothalamic–pituitary–ovarian axis would mirror those for conventional genotypes.

Consistent with our previous work [11], in the present study, we observed a positive effect of Suinfort^®^ on sow fertility although, in this study, the effect was only significant in the summer and autumn months, possibly due to an amelioration of the effect of heat stress on Iberian sows. Seasonal infertility represents a problem for many pig breeding systems, with heat stress impacting sow ovarian activity and oocyte developmental competence [9,18]. These problems are more evident with extensively or semi-extensively reared animals, in tropical climates, or on farms with limited control of the environment or breeds with limited or no selection [12,13,19]. However, although the analysis of parity effects was not one of the objectives of the present work, we noticed some interesting trends that could be explored in more specific studies. Previous studies have shown that gilts have lower reproductive performance and fertility declines in older sows too [20,21]. Iberian sows could present optimal reproductive performance between 2 and 4 years old, which could be confirmed in specific studies.

The effects of Suinfort^®^ in the Iberian sows are likely attributable to the constituent components. The oxytocin may decrease the retrograde flow of sperm while promoting uterine contractions, helping to establish an optimal oviduct sperm reservoir [22,23,24]. Additionally, sperm motility could be enhanced by the caffeine in Suinfort^®^, since caffeine stimulates the motility of both fresh and cryopreserved sperm [25,26]. While sperm motility may not be necessary for transport to the oviduct passage through the uterotubal junction, this might aid in the process. Furthermore, regardless of season, AI fertility is dependent on the timing of sperm deposition relative to ovulation, and the timing of ovulation relative to estrus detection is affected by season [27]. Therefore, the lecirelin component of Suinfort^®^ may have promoted a better synchronization of ovulation, explaining the improvements noticed in summer and autumn compared with CON pigs [28]. Taken together, improved sperm transport to the oviduct and improved timing of AI relative to ovulation may have enhanced fertility in Suinfort^®^-treated Iberian sows.

## 5. Conclusions

The present data demonstrate that seminal supplementation with Suinfort^®^ improved fertility throughout the year of Iberian sows inseminated with Duroc semen. Further studies should investigate whether Suinfort^®^ can be used to improve fertility in Iberian sows inseminated with Iberian semen and other non-commercial autochthonous breeds.

## Figures and Tables

**Table 1:** Sample size for the pregnancy rate study, grouped by treatment and season.

Group	Spring	Summer	Autumn	Winter
CON	517	703	1175	629
SF	413	511	671	374

**Table 2:** Sample size for the prolificacy study, grouped by treatment and season.

Group	Spring	Summer	Autumn	Winter
CON	491	672	1141	617
SF	400	493	655	369

## Data Availability

The data presented in this study are available on request from the corresponding author. The data are not publicly available due to being part of the records of a private company.

## Supplementary Materials

The following are available online at https://www.mdpi.com/article/10.3390/ani11113176/s1, Figure S1: Frequency distribution of total (a) and live (b) piglets per farrowing in each season for the Control and Suinfort^®^ treatments. AI resulting in no pregnancy were excluded, Figure S2: Frequency distribution of stillborn (a) and mummified (b) piglets per farrowing in each season for the Control and Suinfort^®^ treatments. AI resulting in no pregnancy were excluded.
