# Supplementary material for: The Suinfort® Semen Supplement Counters Seasonal Infertility in Iberian Sows
Source: Animals (Basel). 2021 Nov 6;11(11):3176. doi: 10.3390/ani11113176 (PMC8614245; doi:10.3390/ani11113176)
Supplement: Supplementary file 1 [file animals-11-03176-s001.zip › animals-1440942-supplementary.pdf]

animals

MDPI

The Suinfort® Semen Supplement Counters Seasonal Infertility in Iberian Sows

Javier Piñán [1], Felipe Martinez-Pastor [1,2], Beatriz Alegre [1,3], Magdalena Maj [4], Roy N Kirkwood [5], Juan Carlos Domínguez [1,3], Rodrigo Manjarín [6,*]

[1] Institute of Animal Health and Cattle Development (INDEGSAL), Universidad de León, 24071 León, Spain
[2] Department of Molecular Biology (Cell Biology), Universidad de León, 24071 León, Spain
[3] Department of Animal Medicine, Surgery and Anatomy (Animal Medicine and Surgery), Universidad de León, 24071 León, Spain
[4] Department of Biological Sciences, California Polytechnic State University, One Grand Ave, Bldg. 10, San Luis Obispo, CA 93407-0255
[5] School of Animal and Veterinary Sciences, University of Adelaide, Roseworthy, SA 5371, Australia
[6] Animal Science Department, California Polytechnic State University, One Grand Ave, Bldg. 10, San Luis Obispo, CA 93407-0255
* Correspondence: felipe.martinez@unileon.es; Tel.: +34 687365362

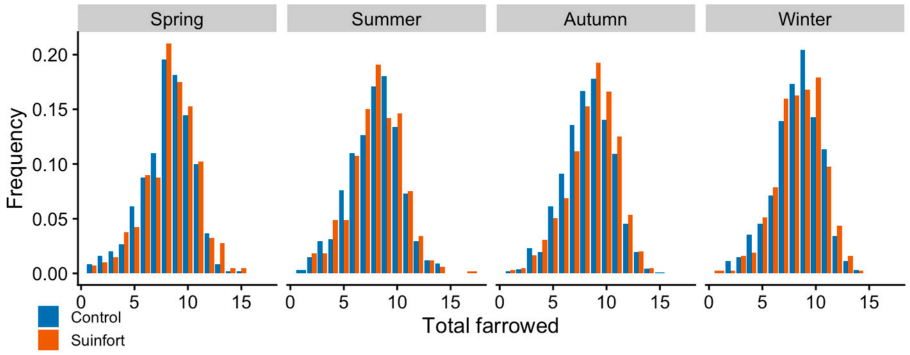

(a) Total piglets born

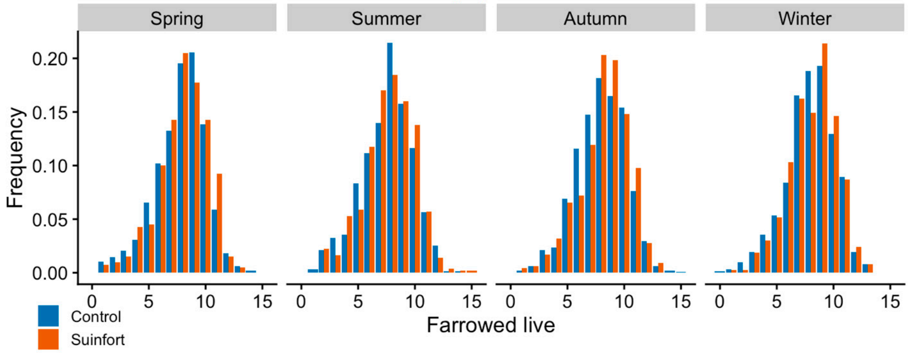

(b) Live born

Figure S1. Frequency distribution of total (**a**) and live (**b**) piglets per farrowing in each season for the Control and Suinfort® treatments. AI resulting in no pregnancy were excluded.

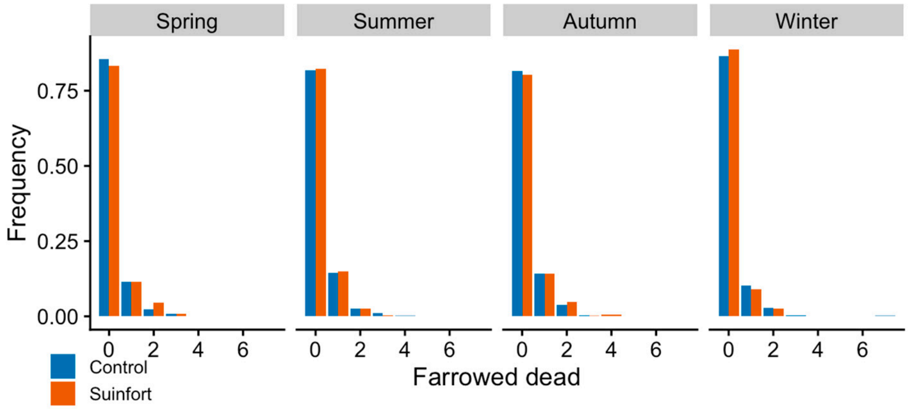

(a) Dead born

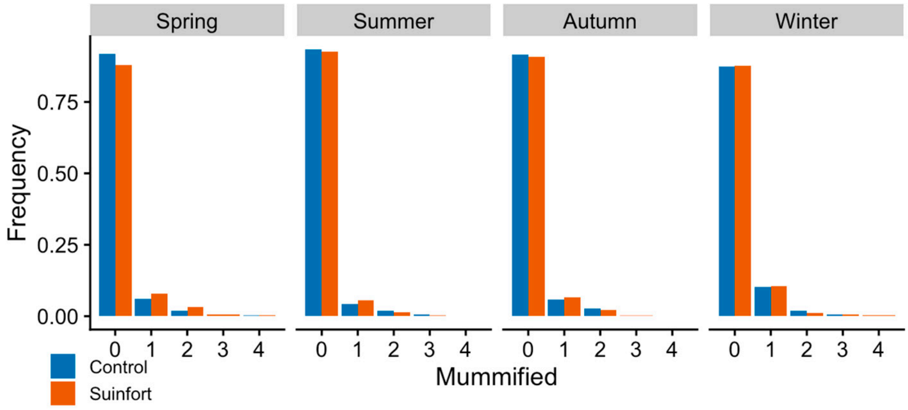

(b) Mummified

Figure S2. Frequency distribution of stillborn (**a**), and mummified (**b**) piglets per farrowing in each season for the Control and Suinfort® treatments. AI resulting in no pregnancy were excluded.
